# Society for Cardiovascular Magnetic Resonance (SCMR) guidelines for reporting cardiovascular magnetic resonance examinations

**DOI:** 10.1186/s12968-021-00827-z

**Published:** 2022-04-28

**Authors:** W. Gregory Hundley, David A. Bluemke, Jan Bogaert, Scott D. Flamm, Marianna Fontana, Matthias G. Friedrich, Lars Grosse-Wortmann, Theodoros D. Karamitsos, Christopher M. Kramer, Raymond Y. Kwong, Michael McConnell, Eike Nagel, Stefan Neubauer, Robin Nijveldt, Dudley J. Pennell, Steffen E. Petersen, Subha V. Raman, Albert van Rossum

**Affiliations:** 1 Division of Cardiology, Department of Internal Medicine, VCU Pauley Heart Center, Virginia Commonwealth University, 1200 East Broad Street, P.O. Box 980335, Richmond, VA 23298 USA; 2 Department of Radiology, University of Wisconsin-Madison School of Medicine and Public Health, Madison, WI USA; 3 Department of Radiology, Medical Imaging Research Center, Leuven, Belgium; 4 Imaging Institute, and Heart and Vascular Institute, Cleveland Clinic, Cleveland, OH USA; 5 Division of Medicine, National Amyloidosis Centre, University College London, London, UK; 6 Departments of Medicine and Diagnostic Radiology, McGill University, Montreal, Canada; 7 The Labatt Family Heart Centre in the Department of Pediatrics, The Hospital for Sick Children, University of Toronto, Toronto, ON Canada; 8 1st Department of Cardiology, Aristotle University, AHEPA Hospital, Thessaloniki, Greece; 9 Departments of Medicine and Radiology, University of Virginia Health System, Charlottesville, VA USA; 10 Department of Medicine, Brigham and Women’s Hospital, Boston, MA USA; 11 Division of Cardiovascular Medicine, Stanford University School of Medicine, Stanford, CA USA; 12 Institute for Experimental and Translational Cardio Vascular Imaging, University Hospital Frankfurt, Frankfurt am Main, Germany; 13 Oxford Centre for Clinical Magnetic Resonance Research, John Radcliffe Hospital, University of Oxford, Oxford, UK; 14 Department of Cardiology, Radboudumc, Nijmegen, The Netherlands; 15 Cardiovascular Magnetic Resonance Unit, Royal Brompton Hospital, London, UK; 16 William Harvey Research Institute, Queen Mary University of London, London, UK; 17 Ohio State University Wexner Medical Center, Columbus, OH USA; 18 Amsterdam University Medical Center, Amsterdam, The Netherlands; 19 Department of Medicine, Heidelberg University, Heidelberg, Germany

These reporting guidelines are recommended by the Society for Cardiovascular Magnetic Resonance (SCMR) to provide a framework for reporting results of cardiovascular magnetic resonance (CMR) examinations. This document builds on previously published guidelines from professional societies (ACC/AHA/ACR/ESC/EACVI and others) [1–3], and is customized here for CMR practice in particular. It is also recognized that the ultimate judgment regarding the propriety of any specific procedure or reporting methodology must be made by the physician or individuals participating within the healthcare delivery system that performs the CMR procedure. An alternative approach that differs from these guidelines, standing alone, does not necessarily imply that the different approach falls below the standard of care. To the contrary, a conscientious provider may reasonably adopt reporting elements different from those set forth in these recommendations when, in the reasonable judgment of the provider, such course of action is indicated by the condition of the patient, limitations of available resources, or a new advancement in knowledge or technology that may occur subsequent to the publication of this document.

Prior to scanning, the SCMR recommends that patients be referred for CMR scans in accordance with Appropriate Use Criteria developed by the SCMR, American College of Cardiology (ACC), American College of Radiology (ACR), American Heart Association (AHA), European Society of Cardiology (ESC) and European Association of Cardiovascular Imaging (EACVI) [4, 5]. The SCMR recommends that scans should be performed in accordance with SCMR developed guidelines for scan acquisition and analysis [6].

The SCMR recommends reporting key elements in all documents including information pertaining to (a) site and equipment information, (b) patient demographics, (c) indications for study, (d) study performance, (e) cardiovascular imaging features of the examination, and f) concluding statements that synthesize the study results into a comprehensive diagnosis that can be used for planning therapy or determining prognosis. If there are prior studies, the date of the prior study and comparison should be performed and included in the report.

The SCMR emphasizes that effective communication is an essential component of any diagnostic imaging procedure for a patient with known or suspected cardiovascular disease. Quality patient care is best achieved when study results are conveyed in a timely fashion to those responsible for treatment decisions. Accordingly, the SCMR recommends that a delivered, finalized report be available, where possible, within two business days of performance of the scan for routine studies, but reported on the same day for patients referred for major acute issues (e.g., suspected pulmonary embolism or aortic dissection).

This document serves as a guide to identify (a) recommended and optional report components, (b) principles used to generate a final report, and (c) suggested communications that may occur other than the final report. A final written interpretation or report should be generated and archived following any CMR examination, procedure, or officially requested consultation to review images regardless of the setting where the CMR scan was performed (hospital, imaging center, physician office, mobile unit, etc.). Within this document, all recommended and optional components are in bold, and the information is provided in outline form.

The general outline for the presentation of the material to be included in a structured CMR report is as follows.

## I. General information (Table 1)

**Table 1:** General information pertaining to the CMR report

	Recommended	Recommended if acquired/if present	Optional
Administrative	Site of service Scanner	N/A	Site ID Accreditation entity and status
Demographics	Unique patient ID Patient date of birth (DOB) or age in years Patient sex Reasons for limited examinations Summary of test findings Physician signature and date (electronic signature and date must be clearly labeled) Adequate description of test	Comparison with previous related studies	Patient Race/Ethnicity
Study referral data	Referring physician	N/A	Referring physician provider number Referring physician specialty
Scheduling and performance of study	Date of procedure Personnel involved in procedure	N/A	Time of procedure
Listing of sequences used	Black-blood Late gadolinium enhancement (LGE) Edema Cine Strain Perfusion Flow Iron MR angiography	N/A	N/A
History and risk factors	Height (in or cm) Weight (lb or kg) Body surface area (BSA) Heart rate (beats per min) Rhythm Blood pressure brachial or femoral (specify which) arterial cuff systolic and diastolic blood pressure (mmHg)	For studies using contrast agents, value and date of the most recent serum creatinine and estimated glomerular filtration rate (eGFR)	N/A
Non-imaging findings associated with examinations			
Studies requiring 12-lead electrocardiogram	Interpretation Rhythm Ventricular rate Presence of Q-waves ST segment T-wave abnormalities	N/A	N/A
Tests incorporation stress testing	Heart rates and rhythm Oxygen saturation Maximum predicted heart rate response for age Each should be recorded during following points in time and more frequently as clinically indicated: at pre-test baseline, at each level of stress, after recovery	N/A	Systolic and diastolic blood pressures
Studies utilizing vasoactive or positive inotrope pharmacological cardioactive agents	Type of agent Quantity of agent Duration of agent Route of administration Associated medications and presence/absence of any side effects	N/A	N/A
Studies utilizing contrast agents	Type and name of contrast agent Volume of agent Route of administration Dosage of agent and presence/absence of any side effects	N/A	N/A
Studies utilizing sedation	General anesthesia Continuous display (hemodynamic or electric) Recording of heart rate and rhythm and/or blood pressure Type, volume, route of administration Any side effects Patient’s cardiovascular and pulmonary response Reason for administration	N/A	N/A
Reporting criteria for CMR examinations	Standardized report format agreed upon in facility Final report reviewed by interpreting member of medical staff Final interpretations verified and signed by medical staff (manual or electronic) within 2 business days of study for routine studies, but reported on the same day for patients referred for major acute issues Permanent record of interpretation Defined mechanism whereby results which demonstrate urgent or life-threatening findings are communicated to appropriate healthcare professionals	If preliminary reports are issued, preliminary nature must be indicated	N/A

### A. Administrative—5 total elements (3 recommended; 2 optional)

1. **Site ID** (recommended): Site ID is a unique number assigned to each study performance site.
2. **Site of service** (recommended): Indicate the type of facility submitting the reporting data. These would include *inpatient hospital, outpatient facility*, *free standing imaging center*, *ambulatory care office*, or *mobile unit*.
3. **Scanner** (recommended): Indicate the type of *magnet*, *manufacturer*, *model number*, *field strength*, and *software platform* of the unit performing the procedure.
4. **Accreditation status** (optional): This should be represented as *yes*, *pending*, or *no*.
5. **Accreditation entity** (optional): For example, (i.e., Intersocietal Accreditation Commission—Magnetic Resonance Laboratories (https://www.intersocietal.org/mri/), etc.).

### B. Demographics—(4 recommended elements)

1. **Unique patient ID:** medical record number used by the health care delivery system where the CMR examination was performed
2. **Patient Date of Birth**
3. **Patient Gender**
4. **Patient Race/Ethnicity**

### C. Study referral data—(2 optional elements)

1. **Referring Physician**—National Provider Identifier (NPI)
2. **Referring Physician Specialty**

### D. Scheduling and performance of study—(6 recommended elements)

1. **Date of procedure**
2. **Procedure start time**
3. **Personnel involved in procedure**
  i. Nursing
  ii. Trainees (e.g., fellows, residents, house officers)
  iii. Staff physicians
  iv. Technologists
4. **Primary indication for test**
5. **Study quality**
6. **Listing of sequences used**
  i. Cine
  ii. Tagged and other strain-encoding cine
  iii. T1- and T2-weighted imaging (T1w; T2w)
  iv. Quantitative T1 and T2 mapping
  v. T2* mapping
  vi. Early gadolinium enhancement
  vii. Late gadolinium enhancement
  viii. Velocity-encoded / phase contrast cine
  ix. MR angiography
  x. Myocardial perfusion

### E. History and risk factors—(3 recommended)

1. **Height**
2. **Weight**
3. For studies using gadolinium-based contrast, value and date of acquisition of the most **recent serum creatinine level** and estimated **glomerular filtration rate** should be included

### F. Non-imaging findings associated with examinations—(5 recommended elements)

1. In those studies requiring 12-lead **electrocardiogram (ECG)**, its **interpretation** should be provided. This includes the rate, rhythm, and *presence of Q-waves*, *ST segment* or *T-wave abnormalities*, or *other rhythm disturbances*.
2. For studies evaluating hemodynamically important conditions (i.e., valvular heart disease, intracardiac shunting, cardiac output, etc.), **heart rates and rhythm**, and **systolic** and **diastolic blood pressure** should be provided during the CMR acquisition. For tests incorporating stress testing, the **heart rates** and **rhythm, oxygen saturation**, **systolic** and **diastolic blood pressures**, and the **percent of maximum predicted heart rate response for age** should all be recorded during the following points in time:
  i. At pre-test baseline
  ii. At each level of stress
  iii. After recovery
3. For studies utilizing vasoactive or positive inotrope pharmacological agents, the **agent, quantity, duration,** and **route of administration of the agents and associated medications** should be provided.
4. For studies utilizing contrast agents, **the type (i.e., paramagnetic), name, dose, and administrative route** should be provided.
5. For studies utilizing sedation, general anesthesia, or supported ventilatory or cardiac (hemodynamic or electrical) assistance, the **amount, type, route and measures of administration of these agents or support** should be documented. Also, the patient’s **cardiovascular and pulmonary response** (heart rate, blood pressure, respiratory rate, and oxygen saturation) should be recorded. The **reason for administration** required of the agent should be provided.

## II. General techniques (Table 2)

**Table 2:** Acquisition methods and procedures for cardiovascular structures

	Recommended	Recommended if acquired/if present	Optional
Left ventricular (LV) structure and function	Method of acquisition Method of analysis LVEDV/LVEDVI LVESV/LVESVI LVSV/LVSVI LVEF CO/CI LVM/LVM index LV end diastolic wall thickness Regional wall motion Aneurysms	N/A	Qualitative assessment of wall thickening
Right ventricular (RV) structure and function	Method of acquisition Method of analysis RVEF Regional wall motion	N/A	RVEDV/RVEDVI RVESV/RVESVI RVSV/RVSVI RV mass Qualitative assessment of wall thickening Curvature of the interventricular septum
First pass perfusion	N/A	First-pass perfusion and late gadolinium enhancement Post MI tissue characterization	N/A
Late gadolinium enhancement	Location Myocardial distribution Extent Interpretation	N/A	Total mass of infarcted tissue
Stress perfusion (vasodilator)	Visual appearance of contrast enhancement Number of segments involved and the suspected etiology of the perfusion defects Extent	N/A	Myocardial perfusion in ml/min/g
Stress function (dobutamine)	Wall motion Inducible LV wall motion abnormalities Contractile reserve	Myocardial perfusion	Wall motion score index
Blood flow	Direction and range of the velocity encoding setting Flow measurements	N/A	Peak and mean velocities and orifice areas
Advanced tissue characterization	*Iron quantification:* septal native myocardial T2*; global native myocardial T1 *Hemorrhage:* regional native myocardial T1; regional native myocardial T2; regional native myocardial T2* *Suspected global acute myocardial injury:* global or regional native myocardial T2; global or regional native myocardial T1 *Fibrosis/infiltration/scar in cardiomyopathies:* global or regional native myocardial T1; *Inflammation − injury* + *edema:* global or regional native myocardial T1; global or regional native myocardial T2	N/A	*Iron quantification:* Global native myocardial T2; global native myocardial T2 *Suspected global acute myocardial injury:* global or regional native myocardial T2 values; global or regional native myocardial T1 values; *Fibrosis/infiltration/scar in cardiomyopathies:* global or regional ECV *Inflammation − injury* + *edema:* global or regional ECV
Strain	Orientation Global or regional extent	Absolute values for peak strain	N/A
Atrial structure and function	Evaluation of left atrial (LA and right atrial (RA) size as within normal limits or dilated Presence of an atrial septal defect Presence of lipomatous hypertrophy of the interatrial septum	N/A	Degree of dilation Left and right atrial volumes and corresponding indices Maximal LA volume Maximal LA volume index to BSA Maximal RA volume Maximal RA volume indexed to BSA Minimal LA volume Minimal RA volume LA longitudinal and transverse dimensions and areas, measured on end-systolic 2, 3, and 4 chamber cine images RA length measured in the end-systolic 4 chamber cine image Evaluation and post-processing recommendations Atrial dimensions or area
Pericardium	Pericardial thickness Pericardial effusion Signs of cardiac tamponade Ventricular (inter)dependence Inflammation of pericardial layers Evidence of associated myocarditis	N/A	Mobility/fusion RV/LV inflow patterns Edema of pericardial layers
MR Angiography	Maximal external diameter, or maximal external perpendicular diameters for asymmetric dimensions For plaque/stenosis: maximum external diameter Location of atherosclerosis and plaque characteristics Thickness/size of atherosclerosis and plaque characteristics Mobility plaques of atherosclerosis and plaque characteristics Estimate % stenosis of atherosclerosis and plaque characteristics if hemodynamically relevant Aortic annulus of thoracic aorta Sinsues of Valsalva of thoracic aorta Sinotubular junction of thoracic aorta Ascending and descending aorta diameters at the level of the pulmonary artery of thoracic aorta Aortic arch diameter of thoracic aorta Comment on sinotubular effacement of thoracic aorta Comment on tortuosity of thoracic aorta Maximal abdominal aortic diameter Number and patency of renal arteries Status of the celiac, superior mesenteric and inferior mesenteric arteries Stent presence/absence; patency/occlusion; presence of thrombus Post-operative/procedural appearance Inflammatory disease of the major vessels Comparison to prior examinations Post-operative appearance	Post-contrast appearance/enhancement of atherosclerosis and plaque characteristics Inflammation of atherosclerosis and plaque characteristics, if sequences acquired Change from prior examinations (include prior dimension and date) Minimal lumen diameters along common femoral arteries, external iliac arteries, common iliac arteries bilaterally	For plaque/stenosis: lumen diameter Cross-sectional area(s), particularly when dimensions are asymmetric Areas and volume measurements of abdominal aorta and peripheral arteries
Valvular assessment	Morphology to be reported as normal or abnormal Qualitative descriptors of function Quantitative findings of function (degree of stenosis or semilunar valves, valve area, degree of stenosis of atrioventricular valves) Degree of valvular regurgitation Severity of any stenosis or regurgitation	N/A	Relevant hemodynamic parameters

*LA*, left atrium/left atrial; *LV*, left ventricle/left ventricular;* LVEDV*, left ventricular end-diastolic volume; *LVEDVI*, left ventricular end-diastolic volume index; *LVEF*, left ventricular ejection fraction; *LVESV*, left ventricular end-systolic volume; *LVESVI*, left ventricular end-systolic volume index; *RA*, right atrium/right atrial; *RVEDV*, right ventricular end-diasolic volume; *RVEDVI*, right ventricular end-diastolic volume index; *RVEF*, right ventricular ejection fraction; *RVESV,* right ventricular end-systolic volume; *RVESVI*, right ventricular end-systolic volume index

In this section, SCMR recommends reporting the orientation in which images are acquired, for example whether a cine stack for the assessment of ventricular volumes was acquired in axial or short axis planes; the pulse sequences used; and whether the data set was two- or three-dimensional. The status of recommended versus optional for each described element in Sect. II is provided relative to the clinical disease specific protocol provided in Sect. III.

Below we provide the recommended and optional elements for each of these components.

### A. Left ventricular (LV) structure and function

1. **LV Volume**
  i. The **method of acquisition** (recommended): should be reported per SCMR Protocol Recommendations document (2).
  ii. The **method of analysis** (recommended): should be reported per SCMR Post-Processing Recommendations documents (1,9).
  iii. Measurements (6 recommended): should be reported relative to normal values for acquisition technique and field.
    - **LV end-diastolic volume (LVEDV**): defined as the total volume of blood (ml) in the LV after maximal filling.
    - **LV end-systolic volume (LVESV):** defined as the remaining volume of blood (ml) in the LV after maximal emptying.
    - **LV stroke volume (LVSV):** defined as the amount of blood (ml) pumped per heart beat (LVESV subtracted from LVEDV).
    - **LV ejection fraction (LVEF):** defined as the fraction or percentage (%) of blood that that leaves the LV during systole **(LVSV/LVEDV).**
    - **LV cardiac output (LVCO):** defined as LVSV multiplied by heart rate.
    - **LV mass (LVM):** defined as the LV myocardial volume multiplied by 1.05 reported in grams (g).
  iv. Indexed LV volumes (5 recommended): Method of indexing per SCMR Post-Processing Recommendations document (1,9), relative to normal values for acquisition technique and field strength. Note that values may be indexed to height as well.
    - **LVEDV index** = LVEDV/body surface area (BSA)
    - **LVESV index** = LVESV/BSA
    - **LVSV index** = LVSV/BSA
    - **LVCO index** = LVCO/BSA
    - **LVM index** = LVM/BSA
  v. **Wall thickness** (recommended): should be reported as *thin*, *hypertrophied*, or *normal*.
    - LV hypertrophy can be described as *concentric*, *eccentric* or *asymmetric*.
2. LV Wall Motion
  i. **Regional wall motion** (recommended): should be described according to the 17-segment model. It is recommended that each segment be classified qualitatively as *normal*, *hyperkinetic, hypokinetic, akinetic, dyskinetic, dyssynchronous, not evaluable due to artifact, or not assessed.*
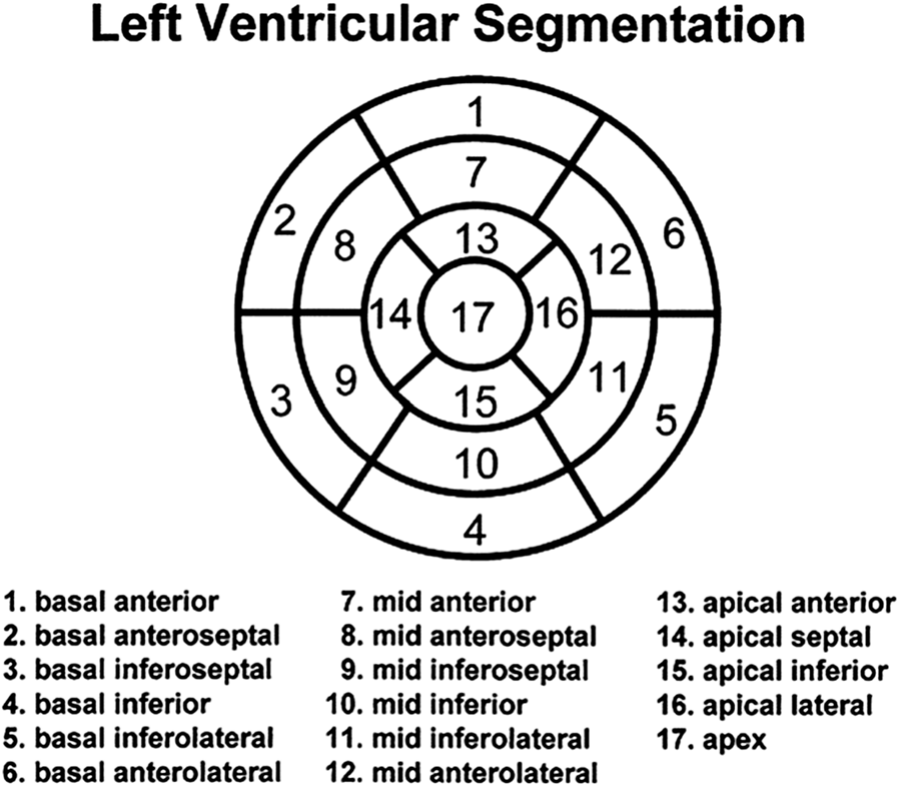
  ii. **Quantitative measurement** (optional): wall thickening or strain per segment
  iii. **Aneurysms** (recommended)**:** need to be recognized as large dyskinetic segments, areas of *multiple* or *discontinuous* segments, and should be defined as *true* or *false* to include information pertinent to surrounding structures

### B. Right ventricular (RV) structure and function

1. RV volume
  i. The **method of acquisition** (recommended): should be reported per SCMR Protocol Recommendations document (2).
  ii. The **method of analysis** (recommended): should be reported per SCMR Post-Processing Recommendations document (1,9).
  iii. Measurements (6 optional): should be reported relative to normal values for acquisition technique and field.
    - **RV end-diastolic volume (RVEDV**): defined as the total volume of blood (ml) in the right ventricle after maximal filling.
    - **RV end-systolic volume (RVESV):** defined as the remaining volume of blood (ml) in the right ventricle after maximal emptying.
    - **RV stroke volume (RVSV):** defined as the amount of blood (ml) pumped per heart beat (RVESV subtracted from RVEDV).
    - **RV ejection fraction (RVEF):** defined as the fraction or percentage (%) of blood that that leaves the RV during systole (RVSV/RVEDV).
    - **RV cardiac output (RVCO):** defined as RVSV multiplied by heart rate.
    - **RV mass (RVM):** The reporting of RVM may be indicated in states of RV hypertrophy, such as hypertrophic cardiomyopathy or pulmonary hypertension.
  iv. Indexed RV volumes (5 recommended): Method of indexing per post-processing document (1,9), relative to normal values for acquisition technique and field strength. Indexing to height may also be done.
    - **RVEDV index** = RVEDV/BSA
    - **RVESV index** = RVESV/BSA
    - **RVSV index** = RVSV/BSA
    - **RVCO index** = RVCO/BSA
    - **RVM index** = RVM/BSA
  v. **Wall thickness** (recommended): should be reported as *thin*, *hypertrophied*, or *normal*.
    - **Right ventricular hypertrophy** can be described as *concentric, eccentric or asymmetric.*
2. RV wall motion
  i. **Regional wall motion** (recommended): should be described qualitatively at the apex, septal and anterior wall and midventricular levels of the RV free wall. Regions should be identified as *normokinetic, hyperkinetic, akinetic, dyskinetic, dyssynchronous, not evaluable,* or *not assessed.*
  ii. **Quantitative measurement** (optional): wall thickening or strain per segment
3. **Curvature of the interventricular septum** (optional): the position of the interventricular septum is a marker of RV pressure and volume load and should be described (separately for end-diastole and peak systole) as *normal (right convex), flat, or bowing into the LV.*

### C. Masses (5 recommended elements)

1. **Location**
2. **Size**
3. **Relationship with other structures**
4. **Criteria for malignancy**
5. **First-pass perfusion and LGE**, if performed
  i. *Present* or *absent*
  ii. Pattern (*homogenous* or *heterogeneous*)

### D. Late gadolinium enhancement (LGE)

1. **Location** (recommended): Reference location of segments as per Section II.A. (LV structure and function) and section II.B. (RV structure and function).
2. **Intramural distribution** (recommended): The intramural distribution of LGE should be classified as adjacent to the epicardium **(***subepicardial***)**, within the substance of the walls *(intramural),* adjacent to the endocardium *(subendocardial),* or spanning the entire segment *(transmural)*
3. **Extent** (recommended): Within each of the 17 LV segments, the transmural extent of enhancement within that segment should be described using the following incremental values:
  i.  ≤ *25%*
  ii. *26% to* ≤ *50%*
  iii. *51% to* ≤ *75%*
  iv. *76% to 100%*
4. **Total mass of LGE tissue** (optional): should be measured in *grams* (g) and also be reported as a percentage relative to the total myocardial mass.
5. **Pattern** (recommended): The appearance of the enhancement should be described as:
  i. *Focal*, in which the pattern appears in one or a few non-contiguous areas
  ii. *Multifocal*, in which the pattern appears in multiple areas, possibly adjacent to one another
  iii. *Diffuse*, in which the pattern appears dispersed and perforated throughout multiple segments.
6. **Interpretation** (recommended): the pattern should be interpreted as indicative of
  - Ischemic injury (myocardial infarction)
  - Nonischemic injury or infiltration (myocarditis, hypertrophic cardiomyopathy, sarcoidosis, etc.)

### E. Vasodilator stress perfusion

At present, the majority of vasodilator stress perfusion studies qualitatively describe the transit of gadolinium contrast through the LV and RV myocardial tissue. As described in the non-imaging findings component of the reported list above, parameters such as vital signs, medications, and contrast agent administration should be reported. We recommend the reporting of LV myocardial information in the format of a 17-segment model through the use of a chart, table, or polar maps (so called “Bull’s Eye” plot).

1. For each LV or RV myocardial segment, the visual appearance of **contrast enhancement** (recommended) within that segment should be characterized as:
  i. *Present* or *Absent*
    - Additionally, after these assessments, *the number of segments* involved and the suspected *etiology* of the perfusion defects should be identified.
  ii. *Persistence:* defined as present for ≤ 5 heart beats, > 5 but ≤ 10 heart beats, or > 10 heart beats.
2. **Quantitative** (optional): myocardial perfusion in ml/min/g

### F. Stress function (dobutamine or exercise)

As described in the non-imaging findings component of the reported list above, parameters such as vital signs, medications, and contrast agent administration should be reported. We recommend the reporting of LV myocardial information in the format of a 17-segment model through the use of a chart, table, or polar maps (so called “Bullseye” plot).

1. Wall Function (4 recommended, 1 optional)
  i. **Wall motion** (recommended): should be described *qualitatively* (wall motion as listed above in the left and right ventricular wall motion descriptions) or *quantitatively* (referenced measure such as % wall thickening, or strain) during testing.
  ii. **Wall motion score index** (optional): *defined as the sum of the wall motion scores divided by the number of segments scored* should be reported at each level of stress.
  iii. **Inducible wall motion abnormalities** (recommended): defined as an *increment in the severity of wall motion* (for example normal at rest to hypokinetic at stress) should be identified within each of the 17 myocardial segments. Also, identifying *failure of global LV* function to improve or worsen during stress should occur.
  iv. **Contractile reserve** (recommended): defined as *improvement in wall motion assessment* (for example akinesis improving to hypokinesis) during low levels of dobutamine infusion should be identified in each of the 17 myocardial segments when any LV dysfunction is present at rest.
  v. **Myocardial perfusion** (acquisition optional, but reporting recommended once acquired): The SCMR suggests that perfusion in each of the 17 segments be defined according to the *transmurality and persistence of the defect*. The committee recommends that stress-induced (exercise or inotropic) perfusion defects be compared with co-registered rest perfusion or late enhancement segments in order to identify *ischemic, infarcted, or non-ischemic areas*. We also recognize that artifacts may mimic defects. These should be described.

### G. Flow

When quantitative flow measurements are acquired, the following parameters are recommended:

1. **Direction of the velocity encoding** (optional): should be reported as *anterior to posterior, superior to inferior, right to left, through plane or a combination thereof*.
2. **Range of the velocity encoding, or Venc setting,** (recommended): *meters/sec*.
3. **Flow measurements** (recommended): *milliliters or liters, per heartbeat or per minute*
4. **Peak and mean velocities and orifice areas** (recommended): with *velocity measurements in m/sec and area results in cm2*. When appropriate, **peak and mean velocities** should include corresponding estimates of peak and mean pressure gradients calculated according to the modified Bernoulli Equation [7].

### H. Advanced tissue characterization

In addition to the standard sequences for LGE, other sequences, including edema-sensitive T2-weighted, iron- and hemorrhage-sensitive T2*-weighted, and early gadolinium enhancement, as well as myocardial T1 mapping, may be used for detecting myocardial tissue pathology [8]. For common suspected pathologies, we recommend reporting the following parameters in addition to standard parameters. Note that it is important that the field strength of the magnet (I.A.iii.) be identified when reporting myocardial T1 values.

1. Iron Quantification (2 recommended, 2 optional)
  i. Septal native myocardial T2* should be reported as *decreased or normal* + value.
  ii. **Septal native myocardial T1** should be reported as *decreased or normal*.
  iii. Global native myocardial T2* (optional)
  iv. **Global native myocardial T1** (optional)
2. Hemorrhage (3 recommended)
  i. **Regional native myocardial T1** should be reported as *decreased or normal*.
  ii. **Regional native myocardial T2** should be reported as *decreased or normal*.
  iii. Regional native myocardial T2* should be reported as *decreased or normal*.
3. Edema-sensitive T2-weighted CMR: The signal intensity relative to the signal intensity in skeletal muscle can be reported. When assessed, it is recommended that the location of increases in T2 signal intensity be identified relative to the 17 segment model.
4. Early gadolinium enhancement: Within the first minutes of the first pass of intravenous gadolinium chelates administration, areas of early enhancement or gradient echo inversion recovery (or comparable imaging sequence) images may be appreciated. The location of this enhancement within the 17-segment model should be identified when acquired.
5. Global or regional edema in suspected global acute myocardial injury (2 recommended, 2 optional)
  i. **Global or regional native myocardial T2** should be reported as *increased or normal***.**
  ii. **Global or regional native myocardial T1** should be reported as *increased or normal*.
  iii. **Global or regional native myocardial T2 values** (optional)
  iv. **Global or regional native myocardial T1 values** (optional)
6. Global or regional fibrosis/infiltration/scar in cardiomyopathies (1 recommended, 1 optional)
  i. **Global or regional native myocardial T1** should be reported as *increased or normal***.**
  ii. **Global or regional ECV** (extra cellular volume fraction) should be reported as *increased or normal* and the absolute value (%) provided (optional)
7. Global or regional myocardial inflammatory injury and edema (2 recommended, 1 optional)
  i. **Global or regional native myocardial T1** should be reported as *increased* or *normal***.**
  ii. **Global or regional native myocardial T2** should be reported as *increased* or *normal***.**
  iii. **Global or regional ECV** should be reported as *increased* or *normal* and the absolute value (%) provided (optional)

### I. Strain

LV myocardial strain values are optional to report. When reported, the following information is suggested.

1. Method of **acquisition** (source images)
2. **Orientation**
  i. *Circumferential, longitudinal, radial*
3. Regional **distribution**
  i. Global or regional extent and severity, absolute values for peak strain or strain rate can be reported (optional)

### J. Atrial structure and function

1. The following qualitative variables should be reported (3 optional, 3 recommended):
  i. **Evaluation of left atrial (LA) and right atrial (RA) size as normal or dilated** (recommended)
  ii. **Degree of dilation classified as** *mild, moderate, severe* (optional)
  iii. **LA and RA volumes and corresponding BSA indices** (optional)
  iv. **Presence of patent foramen ovale or atrial septal defect**, if identified (recommended).
  v. **Presence of lipomatous hypertrophy of the interatrial septum**, if identified (recommended).
  vi. **LA function** (optional)
2. If quantified, **maximal LA volume** (in ml) should be calculated during onset of ventricular systole on the last cine image prior to opening of the mitral valve. The minimal LA volumes should be defined as the first cine image after closure of the mitral valve. Maximal and minimal RA volumes should be calculated using corresponding cine images of the tricuspid valve.
  i. **Maximal LA and RA volumes and indexed for BSA** (optional);
  ii. **Minimal LA and RA volumes and indexed for BSA** (optional);
  iii. **Reporting of LA and RA longitudinal and transverse dimensions and areas, measured on 2, 3, and 4 chamber cine images** (optional).
  iv. Imaging technique used for measurements, as per the SCMR Evaluation and Post-Processing Recommendations document (1,9) (recommended) (e.g., modified Simpson’s method, biplane area-length method, multi-slice disk summation, or qualitative):
  v. **Reporting of LA and RA ejection fraction calculated from maximal and minimal volumes** (optional)
  vi. Reporting of LA and RA stroke volumes and their respective indices calculated from maximal and minimal volumes (optional).

### K. Pericardium—when evaluating the pericardium, the following should be reported

- **Pericardial thickness** (T1w dark blood, cine) (recommended) in *mm*
- **Pericardial effusion** (T1w dark blood, cine) (recommended) in *circumferential or loculated*
- **Mobility/fusion** of pericardial layers (optional)—CMR tagging
- **Signs of cardiac tamponade physiology**—diastolic collapse RV wall / early systolic collapse RA wall (recommended)
  1. May be intermittent (real-time cine during free breathing)
- **Ventricular (inter)dependence**—real-time cine during deep inspiration (recommended)
  1. Early-diastolic inspiratory septal inversion/flattening
  2. Total respiratory-related ventricular septal shift
- **RV/LV inflow patterns**—in case of constrictive pericarditis (optional)
  1. *Inflow patterns* atrioventricular valves / caval and pulmonary veins
  2. *Real-time cardiac inflow* during free breathing
- **Edema of pericardial layers**, i.e., high signal on T2 W images (optional)
- **Inflammation of pericardial layers**, i.e., high signal on LGE images if gadolinium administered (recommended)
- **Evidence of associated myocarditis,** non-ischemic myocardial LGE or edema on T2w / increased values on T1-T2 mapping (recommended)

### L. Cardiac and paracardiac masses, including thrombi

- The **presence***,* **number**, **location** and **extent** of cardiac and paracardiac masses should be reported (recommended).
- Functional questions relate to:
  1. **Extent of myocardial infiltration** (recommended). Tagged cine images may help define a mass as infiltrating the **myocardium** or **pericardial space** (optional)
  2. **Impact** on various aspects of cardiac function such as **myocardial deformation** and **valvular competency** (recommended)
  3. Routine measures of **ventricular size** and **systolic function** should be included from cine imaging in standard cardiac planes (e.g., *horizontal long axis, short axis stack,* etc.), and additional cine planes may help delineate the functional impact of a cardiac or paracardiac mass. (recommended)
  4. The **enhancement** of a mass on first-pass perfusion imaging endorses vascularity or communication with the vascular space; additional clues from morphologic imaging may help distinguish results of first-pass perfusion imaging. (recommended)
  5. Tissue Characterization
    i. Description of main signal intensity characteristics in T1w, T2w, LGE, and LGE/long TI images
    ii. Suspected predominant tissue composition, (e.g., fibrous, fatty, edema, necrotic core), if possible

### M. MR angiography—for the vessels of intent, the following should be reported

1. Dimensions—Maximal **diameter, or perpendicular diameters** for asymmetric dimensions, measured from planes perpendicular to the vessel centerline. Area may be reported as well. There is no consensus in the literature regarding whether diameters are to include lumen or aortic wall since this depends in part on the application and CMR/ MR angiographic technique. In cases of aneurysm and plaque/stenosis, maximum external diameter and lumen diameter may both be reported. We refer to the SCMR Post-Processing Recommendations (1,9) document for further details [9]. Indicate change from prior examination(s), including prior dimensions and date(s).
2. Atherosclerosis and plaque characteristics—The following should be reported:
  i. **Description** of location
  ii. **Mobility** and **extent** of plaques
  iii. **Estimate % diameter stenosis** if hemodynamically relevant
  iv. Post-contrast **appearance**/**enhancement** (if sequences acquired)
3. Flow: On CMR scans of the aorta in which phase-contrast (PC)-CMR or 4D-flow measures are obtained, *the direction* and *magnitude of flow* should be provided. In situations involving dissections, flow in *the true and false lumens* should be reported.
4. Inflammation: Findings on T1w- or T2w images should be reported if the sequences are acquired. Presence/absence of contrast enhancement should also be reported if sequences were performed.

## III. Disease-specific CMR protocols

Upon confirming the appropriateness of an ordered CMR procedure, it is incumbent upon the interpreting physician to report on one or more recommended elements pertinent to the management of the disease process evaluated during the CMR procedure. As such, the SCMR recommends reporting the following data elements that pertain to each of the clinical scenarios listed below and categorized in the following sections (Table 3): Anatomical/Structural, Functional, Perfusion, Tissue Characterization, and Clinical features related to the disease, such as duration of symptoms from onset to CMR / revascularization, duration of time from revascularization to CMR, prior revasculariation (type, localization), coronary and bypass status (open, significant stenosis, occluded).

**Table 3:** Cardiovascular disease specific protocols

Ischemic heart disease
Acute myocardial infarction (MI) or acute coronary syndromes
Chronic ischemic heart disease and viability
Cardiomyopathies and myocardial inflammation
Myocarditis
Hypertrophic cardiomyopathy
Arrhythmogenic RV cardiomyopathy (ARVC)
Hypertensive heart disease
Left ventricular non-compaction
Dilated cardiomyopathy
Iron overload cardiomyopathy
Restrictive cardiomyopathy (e.g., cardiac amyloidosis)
Cardiac sarcoidosis
Cancer-related cardiomyopathies
Recreational drug-induced cardiomyopathies
Heart transplantation
Vascular disease (thoracic aorta)
Aortic aneurysm
Aortic dissection, intramural hematoma, and penetrating ulcer
Post-operative appearance
Inflammatory diseases of the aorta
Congenital disease
Vascular disease (coronary arteries)
Coronary artery anomalies
Aneurysms, coronary arteriovenous malformations, and coronary fistulae
Vascular disease (venous)
Stenosis or thrombosis
Pulmonary veins pre-post ablation
Valvular heart disease
LVEDV, LVEDVI, LVESV, LVESVI, LVSV, LVSVI, LVEF, RVEDVI, RVESVI, RVSVI, and RVEF
Degree of stenosis of atrioventricular valves

### A. Ischemic cardiomyopathies

#### i. Acute MI or acute coronary syndromes

- Anatomical/Structural (4 recommended)
  1. **LV and RV volumes** at end-diastole and end-systole (absolute and normalized for BSA), aneurysms
  2. **LVM**
  3. **Thrombi** (*localization, size*), if present
  4. **Pericardium**
- Functional (3 recommended)
  1. **LVEF and RVEF, stroke volume,** and **cardiac output**
  2. **Valvular function** (*mitral, aortic*)
  3. **Abnormal wall motion**, if present, for each of 17 segments (*hyperkinetic, hypokinetic, akinetic, dyskinetic*)
- Perfusion (1 recommended)
  1. **Microvascular obstruction (MVO)** should be reported if assessed with first pass perfusion
- Tissue Characterization (2 recommended, 2 optional)
  1. **Early enhancement** (optional): *presence, location, extent, segmental transmurality, optional: % LVM, % LGE*
  2. **LGE** (recommended): *presence, location, type, extent, segmental transmurality, optional: % LVM, relation of LGE to wall motion (i.e. hypokinetic segment without any LGE) and interpretation concerning hibernation; pericardial enhancement*
  3. **Intramyocardial hemorrhage** (recommended): *presence, location, extent*
  4. **T2 imaging or mapping** (optional): *presence, location, extent including T2 increase beyond LGE, % of LVM.*
- Other (4 optional)
  1. **Duration of symptoms** from onset to CMR / revascularization
  2. **Duration of time from revascularization to CMR**
  3. **Prior revascularization** (*type, localization*)
  4. **Coronary artery bypass graft (CABG) status** – if available: *open, significant stenosis, occluded*
- Stress Perfusion
  1. Pertinent general information from Section I (recommended)
  2. Pertinent stress perfusion from Section II (recommended)
- Stress wall motion
  1. Pertinent general information from Section I (recommended)
  2. Pertinent stress wall motion from Section II (recommended)

#### ii. Chronic ischemic heart disease and viability

- Anatomical/Structural (5 recommended)
  1. **LV and RV volumes** at end-diastole and end-systole (absolute and normalized for BSA), aneurysms, wall thinning, remodeling
  2. **LVM**
  3. **Intraventricular thrombi** (size, location, shape as layer vs protruding mass)
  4. **Pericardial effusion and / or thickening**
  5. The **presence** and **location** of pleural effusions should be reported.
- Functional (3 recommended)
  1. **LVEF and RVEF,** cardiac output and stroke volume
  2. **Valvular function** (mitral, aortic, tricuspid)
  3. **Wall motion** for each of the 17 segments (hyperkinetic, normokinetic, hypokinetic, akinetic, dyskinetic)
- Tissue Characterization (2 recommended, 4 optional)
  1. **Presence of lipomatous metaplasia** (optional) from T1w images or LGE prior to contrast administration
  2. **Acute vs chronic infarct** from T2w images (optional)
  3. **LGE** (recommended): presence, location, size and intramural extent
  4. **LGE % transmural extent** for affected myocardial segments in case of myocardial dysfunction subtended by a flow limiting stenosis; (recommended)
  5. **Information on prior revascularization** (percutaneous coronary intervention (PCI) / CABG) and angiographic results (optional)
- Stress Perfusion
  1. Pertinent general information from Section I (recommended)
  2. Pertinent stress perfusion from Section II (recommended)
- Stress wall motion
  1. Pertinent general information from Section I (recommended)
  2. Pertinent stress wall motion from Section II (recommended)

### B. Non-ischemic cardiomyopathies

#### i. Myocarditis

In addition to the standard sequences for myocardial tissue characterization (edema-sensitive T2w, iron- and hemorrhage-sensitive T2*-weighted, early gadolinium enhancement, and LGE), myocardial mapping is increasingly used for detecting myocardial inflammation. Recently, the expert consensus recommendations were updated and include the following targets for detecting non-ischemic inflammation [11]:

- Anatomical/Structural
  1. LV morphology (all recommended)
    i. **LVEDV and LVEDVI**
    ii. **LVESV and LVESVI**
- Functional (4 recommended, 1 optional)
  1. **LVEF**
  2. **RVEF**
  3. **LVCO**
  4. **LV cardiac index**
  5. Regional LV **wall motion score** (optional)
- Tissue Characterization
  1. **Edema**: Global or regional signal intensity increase in T2w images (*present vs. absent* or *global or regional* native myocardial T2 *increased vs. normal*) with location
  2. **Inflammatory/post-inflammatory injury/scar: Regional or native T1** (*increased vs. normal*) or **regional LGE** in a non-ischemic regional distribution (*present or absent*) or Global or regional ECV (*increased vs. normal*)
- Other (1 recommended, 3 optional)
  1. Wall motion **score index** (optional)
  2. Pericardial effusion (recommended)
  3. RVEDV, RVEDVI, RVESV, RVESVI, RVEF (optional)
  4. LVM index (optional)

#### ii. Hypertrophic cardiomyopathy

- Anatomical/Structural (all recommended)
  1. **Anatomy, pattern of hypertrophy**
    i. *Predominant regional distribution of hypertrophy (e.g. apical, septal, mid-cavity), if asymmetric*
    ii. *Reverse septal curvature*
    iii. *Mid-cavity or LV outflow tract (LVOT) obstruction*
    iv. *Presence* and *location* of clefts or crypts
    v. *Involvement of RV*
  2. **Severity** of hypertrophy (measurement on short-axis balanced steady state free precession (bSSFP) cine)
    i. *Maximal wall thickness*
    ii. *Thickness of non-hypertrophied segments*
  3. **Papillary muscle anatomy**
    i. Hypertrophied or not
    ii. Apical displacement
  4. **Intraventricular thrombus**
    i. Size, location, mobility of the thrombus
  5. **LV volume**
    i. LVEDV, LVEDVI, LVESV, LVESVI
    ii. LVM, and LVM index
  6. **RV volume**
    i. RVEDV, RVEDVI, RVESV, RVESVI
  7. **Atrial size**
    i. LA diameter on 3-chamber view of the LV
    ii. LA volume
  8. **Apical aneurysm,** if present
- Functional (all recommended)
  1. **LVEF**
  2. **RVEF**
  3. **Systolic anterior motion** (SAM) of the mitral valve leaflets or chordae
    i. Turbulence on LVOT on LV 3-chamber view cine images
    ii. Peak systolic LVOT velocity/gradient (note that peak gradient may be underestimated on CMR compared to Doppler echocardiography due to technical reasons)
  4. **Mitral regurgitation**
    i. Eccentric jet or not, describe mechanism of mitral regurgitation e.g. association with valvular SAM
    ii. Quantification of regurgitant volume and fraction
- Perfusion (optional)
  1. **Vasodilator stress perfusion**
    i. In patients with chest pain to identify microvascular ischemia and differentiate from ischemia due to epicardial coronary artery disease
- Tissue Characterization (recommended-if acquired)
  1. Regional fibrosis on LGE (presence, extent, location, intramural regional distribution pattern
  2. **Advanced tissue characterization indexes** (3 recommended - if acquired, 4 optional)
    i. Native T1 for assessed myocardial segments (optional)
    ii. Location of abnormal T1 (recommended - if acquired)
    iii. *Location and magnitude* of regional T2 increase in patients with troponin rise (recommended - if acquired)
    iv. *ECV* in assessed myocardial segments (optional)
    v. Global ECV (optional)
    vi. *Location and magnitude (%)* of ECV elevation (recommended - if acquired)

#### iii. Arrhythmogenic right ventricular cardiomyopathy (ARVC)

In addition to comments specified in other components of these reporting guideline for LV and RV function, the CMR report for ARVC should specifically comment on the following elements of the ARVC Task Force criteria [10]:

- Anatomical/Structural (all recommended)
  1. **LVEDV, LVEDVI, LVESV, LVESVI, LVM, LVM index**
  2. **RVEDV, RVEDVI, RVESV, RVESVI**
  3. **RV wall motion abnormalities** (RV akinesia, dyskinesia, or dyssynchronous contraction, and if present, location of the wall motion abnormality.
- Functional (all recommended)
  1. **LVEF**
  2. **RVEF**
- Tissue Characterization (all optional)—Secondary imaging features that either support the diagnosis of ARVC or that lead to alternative diagnoses (e.g., sarcoidosis, myocarditis) should also be mentioned. These include:
  1. The **presence** and **location** of LGE (e.g*., RV, LV—ischemic or nonischemic pattern*)
  2. The **presence** and **location** of abnormal T1 that may suggest fatty infiltration
  3. The **presence** and **location** of myocardial edema (if T2w images are acquired)

#### iv. Hypertensive heart disease

- Anatomical/Structural (all recommended)
  1. **LV volumes and mass**
    i. *LVEDV, LVEDVI, LVESV, LVESVI*
    ii. *LVM, LVM index*
  2. Mean and maximal **wall thickness** (indexed to BSA or height)
    i. *Normal or increased*
  3. **RV volumes**
    i. *RVEDV, RVEDVI, RVESV, RVESVI*
  4. **Atrial size**
    i. *LA diameter* on 3-chamber view of the left ventricle
    ii. *LA volume*
- Functional
  1. **LVEF** (recommended)
  2. **RVEF** (recommended)
  3. **LV regional wall motion abnormalities**
  4. **Mitral regurgitation**
    i. *Central or eccentric*, describe likely **mechanism** of MR, e.g., dilatation of mitral annulus
    ii. Quantification of regurgitant volume and fraction
  5. Assess **aortic valve function** for regurgitation, particularly in patients with dilated aortic root and/or ascending aorta (recommended)
    Assess **thoracic aorta diameters**: *annulus, sinuses, ascending aorta, arch, descending thoracic aorta*
- Perfusion
  1. **Vasodilator stress perfusion** (optional)
    i. in patients with known coronary artery disease to assess for inducible ischemia
    ii. in patients where coronary anatomy is not known to exclude inducible ischemia
- Tissue Characterization
  1. Regional abnormality on LGE (all recommended)
    i. **Presence** of regional abnormality (*yes/no*)
    ii. **Location** of regional abnormality
    iii. **Pattern** of non-ischemic LGE (*midwall, patchy*)
    iv. Describe the **presence** of any co-existing infarct scar
    v. **Extent** of LGE (qualitative assessment of extent)
  2. Advanced tissue characterization indexes (optional)
    i. *Native T1*
    ii. *ECV*

#### v. LV non-compaction

- Anatomical/Structural (all recommended)
  1. **LV volumes**
    i. *LVEDV, LVEDVI, LVESV, LVESVI, LVEF, LVSV*
  2. **RV volumes**
    i. *RVEDV, RVEDVI, RVESV, RVESVI, RVEF, RVSV*
  3. **Excessive trabeculation** in combination with thin solid myocardium:
    i. **Measurement of maximal ratio** between the thickness of the trabeculated endocardial layer and the solid epicardial layer in cross-sectional cine images (recommended).
    ii. **Percentage of LVM** that consists of trabeculation (optional).
    iii. **Systolic rotation of the apex** (optional)
    Iv. **Wall motion abnormalities** of non-compacted segments (recommended)
- Functional (all recommended)
  1. **LVEF**
  2. **RVEF**
- Tissue Characterization
  1. **Myocardial fibrosis**
    i. CMR features: *LGE* of LV myocardium (recommended)
  2. **LV or RV thrombi** in trabecular recesses, if present (recommended)
  3. Advanced tissue characterization
    i. Features of associated phenotypes such as hypertrophic cardiomyopathy and dilated cardiomyopathy (recommended if pertinent images were acquired)

#### vi. Dilated cardiomyopathy

- Anatomical/Structural (all recommended)
  1. **LV volumes and mass**
    i. *LVEDV, LVEDVI, LVESV, LVESVI*
    ii. *LVM, LVM index*
  2. **Wall thickness**
    i. *Preserved, thin-walled* or *increased*
  3. **Trabeculations**
    i. *Normal*
    ii. *Prominent*
    iii. Fulfilling LV non-compaction *criteria*
  4. **RV volumes**
    i. *RVEDV, RVEDVI, RVESV, RVESVI*
  5. **Atria**
    i. *LA diameter* on 3-chamber view of the LV
    ii. *LA volume*
    iii. **Pulmonary vein anatomy** in patients with atrial fibrillation who may need ablation (optional)
- Functional (all recommended)
  1. **LVEF**
  2. **RVEF**
  3. **Regional wall motion abnormalities** (hypokinesis, akinesis, dyskinesis)
  4. **Dyssynchrony**
    i. *Presence and location of dyssynchrony* (recommended)
    ii. Strain-related data (optional)
  5. **Intraventricular thrombus** if present (recommended)
    i. *Size*, *location*, *mobility* of the thrombus
  6. **Mitral regurgitation** (recommended)
    i. *Central* or *eccentric*, describe likely mechanism of mitral regurgitation, e.g., dilatation of mitral annulus
    ii. *Quantification* of regurgitant volume and fraction
  7. *Aortic valve function* / exclude significant aortic regurgitation
- Perfusion
  1. **Vasodilator stress perfusion** (optional)
    i. In patients with known coronary artery disease to assess for inducible ischemia
    ii. In patients where coronary anatomy is not known to exclude inducible ischemia
- Tissue Characterization
  1. Regional fibrosis on LGE images (presence, location, regional distribution pattern) (recommended)
  2. **Extent** of LGE (% of LV mass (optional)
  3. Advanced tissue characterization indexes (optional)
    i. *Native T1*
    ii. *T2 (in patients with troponin rise)*
    iii. *ECV*

#### vii. Iron overload cardiomyopathy

- Anatomical/Structural (all recommended)
  1. **LV volumes and mass**
    i. *LVEDV, LVEDVI, LVESV, LVESVI*
    ii. *LVM*
  2. **RV volumes**
    i. *RVEDV, RVEDVI, RVESV, RVESVI*
  3. **Atrial size**
    i. *LA area* normalized to BSA
- Functional (all recommended)
  1. **LVEF**
  2. **RVEF**
- Tissue Characterization
  i. T2* values of the full width of the interventricular septum (recommended).
  ii. **Native T1** (optional)

#### viii. Restrictive cardiomyopathy, amyloidosis

- Anatomical/Structural (all recommended)
  1. **LV and RV mass and volumes**: *increased LVM, LV wall thickness and concentric remodeling*.
  2. **Atrial size**: *atrial dilatation contraction, atrial wall thickening*.
  3. **Extracardiac findings**: *pericardial and pleural* effusion.
- Functional (all recommended)
  1. **LVEF**
  2. **RVEF**
- Tissue Characterization (all recommended)
  1. **LGE** Presence of diffuse *subendocardial or transmural LGE coupled with abnormally low blood pool signal.* Difficulties in achieving myocardial nulling over a range of inversion times. Quantification of LGE is not recommended.
  2. **Native myocardial T1** (increased vs. normal)
  3. **ECV** (diffusely increased, early marker).
  4. **T2** (increased vs normal)

#### ix. Cardiac sarcoidosis

- Anatomical/Structural (all recommended)
  1. **Presence of regions with significant wall thinning or LV aneurysms**
  2. **LV and RV volumes and mass**:
    i. LVEDV
    ii. LVESV
    iIi. LVESV
    iv. LVM
  3. **LV and RV systolic function**
    i. LVEF, RVEF
    ii. CO, CI
  4. **Atrial size and function:**
    i. Descriptive atrial size and function (recommended)
    ii. LA and RA diameters (optional)
    iIi. LA and RA volumes (optional)
    iv. LA emptying fraction (optional)
  5. Overall **myocardial function and structure** can be normal in a large proportion of patients with proven cardiac sarcoidosis.
- Functional (all recommended)
  1. **LVEF**
  2. **RVEF**
- Tissue Characterization (all recommended)
  1. **Presence, location and extent of LGE abnormalities**
  2. **Pattern of LGE distribution** (co-existence of ischemic and non-ischemic distribution)
  3. **Edema** (T2w sequences / T2 mapping) with *location* and *magnitude* of T2 elevation.

#### x. Cancer-related cardiomyopathies

- Anatomical/Structural (all recommended)
  1. **LV volumes and mass**
    i. *LVEDV, LVEDVI, LVESV, LVESVI*
    ii. *LVM, LVM index*
  2. **Wall thickness**
    i. *Preserved, thin-walled or increased*
  3. **RV volumes**
    i. *RVEDV, RVEDVI, RVESV, RVESVI*
  4. **Atrial size**
    i. *LA diameter* on 3-chamber view of the left ventricle
    ii. *LA volume*
- Functional (recommended)
  1. **LVEF**
  2. **RVEF**
  3. **Regional wall motion abnormalities**
  4. **Vasodilator stress perfusion** (optional)
    i. In patients with known coronary artery disease to assess for inducible ischemia
    ii. In patients where coronary anatomy is not known to exclude inducible ischemia
  5. Pericardial disease (if present)
- Tissue Characterization
  1. **Regional myocardial abnormality by LGE** presence, extent, location, regional distribution pattern (recommended)
  2. **Advanced tissue characterization indexes**
    i. *Native T1* for assessed myocardial segments (optional)
    ii. *Location* and *magnitude* of elevation in T1 (recommended)
    iii. *T2* for assessed myocardial segments in patients with troponin rise (optional)
    iv. *Location and magnitude* of regional T2 increase in patients with troponin rise (recommended)
    v. *ECV* in assessed myocardial segments (optional)
    vi. Global ECV (optional if performed)
    vii. *Location and magnitude (%)* of ECV elevation (recommended if performed)
- Other
  1. Valvular function
    i. **Valvular stenosis**
      - Assess qualitatively the presence of valve thickening (see valvular disease)
      - Report **severity of valve stenosis** by planimetry and continuity methods
      - Report **mean and peak transvalvular gradients**
  2. **Mitral regurgitation**
    i. Central or eccentric, mechanism of mitral regurgitation, e.g. dilatation of mitral annulus, if applicable
    ii. Quantification of regurgitant volume and fraction
  3. **Myocardial masses**
    i. Cardiac or paracardiac tumor
    ii. Intraventricular thrombus
      - Size, mobility, location
      - Perfusion
        qualitative appearance and heterogeneity of perfusion in 2 orthogonal planes

#### xi. Recreational drug-induced cardiomyopathies (cocaine, amphetamine and alcohol)

- Anatomical/Structural
  1. **LV hypertrophy, dilatation** (recommended)
    i. *LVEDV, LVEDVI, LVESV, LVESVI, LVSV, LVM*
  2. RV involvement (**RV dilatation**) (recommended)
    i. *RVEDV, RVEDVI, RVESV, RVESVI, RVSV*
  3. **Vascular involvement**
    i. *Aortic aneurysm, dissection/ hematoma or dissection or aneurysm of the proximal coronary arteries* (optional)
- Functional (recommended)
  1. **LVEF**
  2. **RVEF**
  3. **Regional wall motion abnormalities**
- Perfusion
  1. Inducible myocardial perfusion deficits associated with microvascular disease (optional)
- **Tissue Characterization** (recommended):
  1. **Myocardial abnormality by LGE**
    i. Presence, location, extent, intramural distribution
  2. **Myocardial Edema**
    i. Presence, location, extent

#### xii. Heart transplantation

The following should be reported post-cardiac transplant:

- Anatomical/Structural:
  1. LV and RV **volumes** (recommended) -
  2. LVM mass (optional)
  3. Atrial **size** (recommended)
    i. *Bi-atrial surgical approach*: bilaterally enlarged atria (predominantly the longitudinal dimensions) with “double LA” appearance. Changes in the atrial structure of the transplanted heart are caused almost universally by structural alterations necessitated by surgical implantation.
- Functional (recommended)
  1. **LVEF** and **RVEF**: Similar to patients who have undergone other forms of cardiac surgery, post–cardiac transplant patients often have abnormalities of interventricular septal motion (recommended)
  2. Aortic regurgitation and mitral regurgitation
- Tissue Characterization (recommended)
  1. Infarct-atypical **LGE**.
- Extracardiac findings: **Pericardial effusions**
- Criteria for **primary graft failure** (optional), identified as:
  1. Severe LV systolic dysfunction
  2. Global elevation in T2
  3. Absence of LGE
- Criteria for acute **rejection** (optional)
  1. Acute deterioration in LV function
  2. Increased myocardial native T1 and T2
  3. Increase in ECV
- Criteria for cardiac **vasculopathy** (optional)
  1. *Infarct-typical LGE*
  2. *Reduced myocardial perfusion reserve*
- Criteria for chronic **graft failure** (optional)
  1. Global dysfunction or regional dysfunction
  2. LGE secondary to vasculopathy-related infarction.

### C. Vascular disease (Arterial)

#### 1. Thoracic aorta

i. Dimensions/areas to include (all recommended):
  - *Aortic annulus*
  - *Sinuses of Valsalva*
  - *Sinotubular junction*
  - *Ascending and descending aorta* at the level of the pulmonary artery
  - *Aortic arch—proximal and distal;* comment regarding whether the aorta is right- or left-sided
ii. **Findings** when present (all):
  - Comment on **sinotubular effacement** (recommended)
  - Comment on **tortuosity** (recommended)
  - **Aortic aneurysm**
    1. Maximum *diameter*
    2. *Morphology* (saccular versus fusiform)
    3. *Location* in the aorta
    4. *Relation* to branch vessels
    5. *Presence, size and location* of mural thrombus
    6. Visceral compressive *effects* (effacement, expansion of the aorta against surrounding structures)
    7. *Presence* of aortic valve abnormalities
    8. *Presence* of periaortic, mediastinal, pericardial, or pleural effusion.
  - **Aortic dissection, intramural hematoma, and penetrating ulcer** (recommended)**:**
    1. Dissection *classification* (per either Stanford or DeBakey)
    2. *Presence* of intimal flap, location of tear or areas of communication (if possible)
    3. *Description of the size and extent* of the true and false lumens
    4. *Presence* of thrombus in false lumen
    5. *Description of flow in false lumen*
    6. *Branch vessel involvement*
    7. *Presence* of periaortic, mediastinal, pericardial, or pleural fluid
  - **Post-operative appearance** (recommended):
    1. as above, noting any *graft insertion points and dimensions*.
  - **Inflammatory diseases of the aorta** (recommended):
    1. Aortic wall *thickness*
    2. Tissue *characterization (T2, contrast agent uptake)*
    3. *Branch vessel involvement*
    4. *Presence* of periaortic, mediastinal, pleural, or pericardial fluid.
  - **Congenital disease** involving the aorta and ventriculoarterial connections (recommended):
    1. See separate SCMR Recommendations for Reporting CMR in Congenital Heart Disease document [12].
iii. Plaque characteristics, as described in section II.M.2.
iv. Flow, as described in section II.M.3.
v. Inflammation, as described in section II.M.4.

#### 2. Coronary arteries

- Anatomical/Structural
  1. **Origins of coronary arteries** are *normal or anomalous* (recommended)
    i. If anomalous, report on proximal course of the anomalous vessels
    ii. Refer to SCMR Recommendations on CMR for Congenital Heart Disease document for further details
  2. **Presence** of additional acquired or congenital findings, if present (recommended)
    i. Aneurysms
    ii. Coronary arteriovenous malformations, coronary fistulae
- Tissue Characterization (optional)
  1. Coronary vasculitis
    i. *Presence/absence* of contrast enhancement (if sequences performed)

### D. Vascular disease (Venous) (all recommended)

The **number** and **position** of pulmonary veins should be accounted for with notation of common trunks and accessory pulmonary veins**. Evidence for stenosis or thrombosis** by cross sectional area of the pulmonary vein may be provided. A 3D workstation may be used to calculate **major** and **minor axes**, and/or **cross-sectional area of each pulmonary vein ostium.** Pre- and post-ablation cross-sectional areas and dimensions of the pulmonary veins should be compared side by side.

i. **Qualitative** elements that should be included in CMR-based pulmonary vein reporting include (recommended):
  - **Number** of pulmonary veins, specifically whether there are four pulmonary veins (*right superior, right inferior, left superior, and left inferior*);
  - **Atrial side** (*right or left*) and **position** (*superior or inferior*) of pulmonary vein return;
  - In separate statements, **recognition** of accessory or anomalous pulmonary veins;
  - **Presence** or **absence** of stenosis in each pulmonary vein, especially in reporting post-ablation exams;
  - **Position** of esophagus relative to the closest pulmonary vein.
  - Overall **statement** on whether pulmonary venous return is *normal* or *anomalous*.
ii. **Quantitative** elements that should be included in pulmonary vein reporting are (recommended):
  - **Cross-sectional area** and **maximum ostial dimension** of each pulmonary vein, ideally derived from imaging with two orthogonal planes;
  - **Cardiac phase** (e.g., *end-atrial diastole*) and **respiratory phase** (e.g., *end-expiration*) during acquisition of images used for measurements;
  - **Cross-sectional area** and **minimum ostial dimension** of each stenotic pulmonary vein; and
  - **Imaging technique** used for measurements.

### E. Valvular heart disease

i. Anatomical/Structural (all recommended)
  - **LVEDV, LVEDVI, LVESV, LVESVI, LVSV, LVEF, RVEDVI, RVESVI, RVSV, RVEF**
  - For the valve(s) of interest, **morphology** should be reported as *normal* or *abnormal* with details on any abnormalities (e.g., bicuspid aortic valve, cleft mitral valve, apically-displaced tricuspid annulus with foreshortened septal tricuspid valve leaflet).
ii. Functional (all recommended)
  - For the valve(s) of interest, **qualitative descriptors** of function should be reported as *normal* (e.g., normal excursion of aortic leaflets) or *abnormal* (e.g., restricted opening of aortic leaflets).
  - For the valve(s) of interest, **quantitative findings** of function should be reported, relevant to the pathology.
    1. **Degree of stenosis of semilunar valves**
      i. *peak systolic velocity*,
      ii. *mean gradient*,
      iii. *valve area*, including method used to compute valve area.
    2. **Degree of stenosis of atrioventricular valves**
      i. *mean gradient*
      ii. other *relevant hemodynamic parameters* (e.g., deceleration time in mitral stenosis).
    3. **Degree of valvular regurgitation** should be reported as regurgitant fraction.
  - Any **stenosis or regurgitation** should be described as *mild, moderate* or *severe* based on a collective interpretation of qualitative and quantitative findings (e.g., moderate pulmonary regurgitation, mild aortic stenosis).
iii. Tissue Characterization
  - **Concomitant findings of myocardial pathology** by LGE, T1, and T2 if acquired are recommended.
  - **Quantitative results** when obtained are recommended to report with indications where feasible of the potential underlying mechanism of abnormality (e.g., extensive midwall LGE enhancement or suspected cardiac amyloidosis).
iv. Angiography
  - **Aortic dimensions** when feasible are recommended to report, especially in valvular heart lesions with associated aortopathy (e.g., bicuspid aortic valve).
  - Other **angiographic findings** are recommended to report where relevant (e.g., pulmonary artery dimensions in patients with significant pulmonary regurgitation after tetralogy of Fallot repair; any abnormal findings).
  - Clinical features related to the disease
    1. **Relevant patient history** and **background data** that informed design and interpretation of the CMR examination are optional to report.

## IV. Extra-cardiac findings

A. It is recognized that there may be findings unrelated to the cardiovascular system identified during CMR imaging procedures. Such findings should be reported in accordance with local standards. However, SCMR recognizes that the techniques/contrast, resolution and field of view of a CMR study are optimized for the cardiovascular system rather than to assess for abnormalities outside of the cardiovascular system.

## V. Summary and conclusions

SCMR recommends that each report concludes with appropriate statements that specifically relate the findings to the study indications. We recommend that these statements provide referring physicians with conclusions that allow the prescription of therapy based on the study findings. We recommend that the conclusion of the report provide the written or electronic signature of the individual accomplishing the report along with the time and date of the signature. We consider it optional to provide the Health Care Provider Identifier for the physician signing the report.

## VI. Principles of disseminating the final report

A. The final signed report is considered to be the definitive means of communicating to the referring physician or other relevant health care provider. Other methods of rapid communication are encouraged in certain situations, such as critical findings, unexpected abnormal findings, or findings that may immediately alter the patient’s course of treatment. When urgent findings are communicated, the mode of contact, name, date, and time of contact should be included in the report.
B. The report should be reviewed for interpretive, descriptive, or transcription errors prior transmitting the final results.
C. The final report should be completed in accordance with governmental or health care facility medical records regulations.
D. The signed written report should be immediately transmitted to the referring physician or health care provider who is treating the patient once it has been finalized and in accordance with appropriate governmental requirements.
E. When feasible, a copy of relevant key images should accompany the final report.
F. A copy of the final report should be archived at the imaging facility as part of the patient’s medical record and be retrievable for future reference. Retention and distribution of these records should be in accordance with governmental regulations and facility policies.
G. Communications other than the final report
  1. We strongly encourage the rapid dissemination of a finalized report. It is recognized however, that preliminary reports may be necessary in certain situations. Preliminary reports should be identified as such; however, it is recognized that their accuracy may be limited.
  2. If a change is made by a disseminated preliminary report discrepant from the final interpretation, then written documentation and communication to all treating or referring physicians is indicated.
  3. It is recommended that all methods of such communications be included in the final report.
H. Self-referred and Third-Party Referred Patients: We recognize that some individuals may seek imaging studies as part of a self-referral or referred by a third party, such as an insurer or an employer.
  1. Self-referred patients
    i. Imagers should recognize that performing imaging studies on self-referred patients establishes a doctor-patient relationship that includes responsibility for communicating the results of imaging studies directly to the patient and arranging for appropriate follow-up.
  2. Third-Party Referred Patients
    i. Patients may be referred for imaging studies by insurance companies, employers, research studies, other benefit programs, or in some instances, attorneys. In such cases, the reports of these studies are frequently communicated through their requesting entity to a clinician or directly to the third party designated clinician. The results of these examinations are then communicated to the patient directly. Regardless of the source of the referral, the diagnostic imager has an ethical responsibility to insure communication of unexpected or serious findings to the patients. It is suggested that each imaging organization that desires to scan and generate reports on self-referred or third-party referred patients develop communication policies within their centers to address evolving issues in this arena.

## Data Availability

Not applicable.

## Abbreviations

ACC: American College of Cardiology
ACR: American College of Radiology
AHA: American Heart Association
ARVC: Arrhythmogenic right ventricular cardiomyopathy
BSA: Body surface area
bSSFP: Balanced steady state free precession
CABG: Coronary artery bypass graft
CMR: Cardiovascular magnetic resonance
EACVI: European Association of Cardiovascular Imaging
ECG: Electrocardiogram
ECV: Extracellular volume fraction
ESC: European Society of Cardiology
LA: Left atrium/left atrial
LGE: Late gadolinium enhancement
LV: Left ventricle/left ventricular
LVCO: Left ventricular cardiac output
LVEDV: Left ventricular end-diastolic volume
LVEDVI: Left ventricular end-diastolic volume index
LVEF: Left ventricular ejection fraction
LVESV: Left ventricular end-systolic volume
LVESVI: Left ventricular end-systolic volume index
LVM: Left ventricular mass
LVOT: Left ventricular outflow tract
LVSV: Left ventricular stroke volume
LVSVI: Left ventricular stroke volume index
MI: Myocardial infarction
MVO: Microvascular obstruction
PC: Phase contrast
PCI: Percutanous coronary intervention
RA: Right atrium/right atrial
RV: Right ventricle/right ventricular
RVCO: Right ventricular cardiac output
RVEDV: Right ventricular end-diastolic volume
RVEDVI: Right ventricular end-diastolic volume index
RVEF: Right ventricular ejection fraction
RVESV: Right ventricular end-systolic volume
RVESVI: Right ventricular end-systolic volume index
RVM: Right ventricular mass
RVSV: Right ventricular stroke volume
RVSVI: Right ventricular stroke volume index
SAM: Systolic anterior motion
SCMR: Society for Cardiovascular Magnetic Resonance
T1w: T1 weighted
T2w: T2 weighted
